# Genome-wide Mapping of Transcriptional Start Sites Defines an Extensive Leaderless Transcriptome in *Mycobacterium tuberculosis*

**DOI:** 10.1016/j.celrep.2013.10.031

**Published:** 2013-11-21

**Authors:** Teresa Cortes, Olga T. Schubert, Graham Rose, Kristine B. Arnvig, Iñaki Comas, Ruedi Aebersold, Douglas B. Young

**Affiliations:** 1 Division of Mycobacterial Research, MRC National Institute for Medical Research, Mill Hill, London NW7 1AA, UK; 2 Department of Biology, Institute of Molecular Systems Biology, ETH Zurich, 8093 Zurich, Switzerland; 3 Systems Biology Graduate School, 8057 Zurich, Switzerland; 4 Institute of Structural and Molecular Biology, University College London, London WC1E 6BT, UK; 5 Genomics and Health Unit, Centre for Public Health Research (FISABIO-CSISP), 46020 Valencia, Spain; 6 CIBER in Epidemiology and Public Health, 28029 Madrid, Spain; 7 Faculty of Science, University of Zurich, 8057 Zurich, Switzerland; 8 Centre for Molecular Bacteriology and Infection, Imperial College London, London SW7 2AZ, UK

## Abstract

Deciphering physiological changes that mediate transition of *Mycobacterium tuberculosis* between replicating and nonreplicating states is essential to understanding how the pathogen can persist in an individual host for decades. We have combined RNA sequencing (RNA-seq) of 5′ triphosphate-enriched libraries with regular RNA-seq to characterize the architecture and expression of *M. tuberculosis* promoters. We identified over 4,000 transcriptional start sites (TSSs). Strikingly, for 26% of the genes with a primary TSS, the site of transcriptional initiation overlapped with the annotated start codon, generating leaderless transcripts lacking a 5′ UTR and, hence, the Shine-Dalgarno sequence commonly used to initiate ribosomal engagement in eubacteria. Genes encoding proteins with active growth functions were markedly depleted from the leaderless transcriptome, and there was a significant increase in the overall representation of leaderless mRNAs in a starvation model of growth arrest. The high percentage of leaderless genes may have particular importance in the physiology of nonreplicating *M. tuberculosis*.

## Highlights

• A resource for the identification of in vitro active promoters in *M. tuberculosis*
• A quarter of all genes in *M. tuberculosis* are expressed as leaderless mRNAs
• Leaderless mRNAs are differentially associated with toxin-antitoxin modules
• Abundance of leaderless mRNAs increases during starvation-induced growth arrest

## Graphical Abstract

**Figure:**
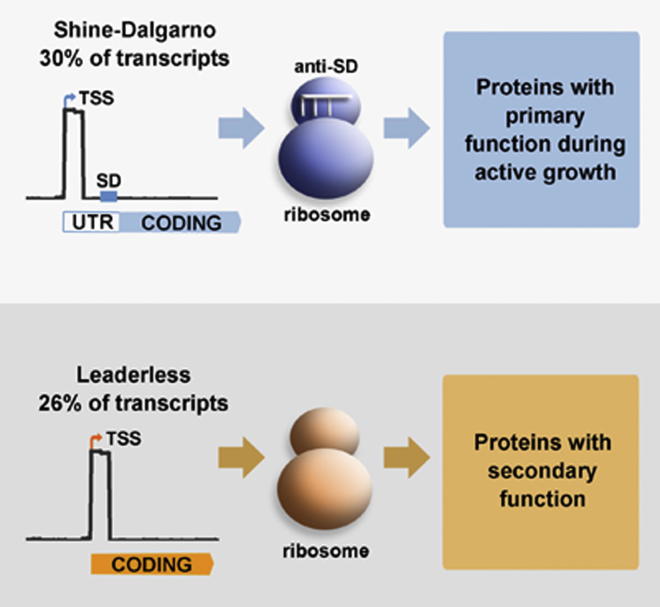

## Teaser

In this study, Cortes, Young, and colleagues report genome-wide mapping of transcriptional start sites combined with RNA sequencing and shotgun proteomics in the human pathogen *Mycobacterium tuberculosis*. A striking finding is a high proportion of genes expressed in the form of leaderless transcripts lacking the Shine-Dalgarno sequence conventionally required for translation. The distribution of functional gene classes between leaderless and Shine-Dalgarno transcriptomes suggests that changes in the specificity of translation may play a role in bacterial adaptation during infection.

## Introduction

*Mycobacterium tuberculosis* is a highly successful human pathogen with a complex life cycle that can involve prolonged periods of asymptomatic infection prior to an active disease process that is required for onward transmission. It is likely that this involves transition of the bacteria between replicating and nonreplicating states in response to environmental changes triggered by immune effector mechanisms and enclosure and escape from granulomatous lesions (Barry et al., 2009). Associated phenotypic changes in tolerance and susceptibility to antimicrobial drugs are thought to contribute to the requirement for prolonged therapeutic regimens to cure tuberculosis.

In the classical *Escherichia coli* paradigm, translation of bacterial mRNA is initiated by binding of a Shine-Dalgarno sequence in the 5′ UTR to the complementary region of 16S rRNA (Shine and Dalgarno, 1974). However, recent bioinformatic analyses suggest that the frequency of Shine-Dalgarno sequences is lower in some eubacterial genera (Nakagawa et al., 2010; Zheng et al., 2011) and that they may resemble Archaea in having a greater reliance on translation of leaderless mRNAs that lack a 5′ UTR (Tolstrup et al., 2000; Slupska et al., 2001; Torarinsson et al., 2005; Brenneis et al., 2007). Leaderless mRNAs are translated with lower efficiency by *E. coli* ribosomes (Moll et al., 2002), though this preference can be altered during exposure to stress (Moll and Engelberg-Kulka, 2012; Moll et al., 2004) or as a result of changes to the ribosome induced by antibiotics or bacterial toxins (Kaberdina et al., 2009; Vesper et al., 2011). In addition to their role in initiation of translation, 5′ UTRs harbor riboswitches and other regulatory elements that can be important for virulence (Wurtzel et al., 2012). The Actinobacteria (which include *M. tuberculosis*) are characterized by a particularly low frequency of genes with predicted Shine-Dalgarno sequences (Ma et al., 2002), and several studies have reported individual instances of leaderless mRNAs in *M. tuberculosis* (Donà et al., 2008; Forse et al., 2011; Chang et al., 2012, Hotter et al., 2008), but the overall number of these transcripts as well as their potential implication in *M. tuberculosis* replicative/nonreplicative programs remains unknown.

Adaptive responses of *M. tuberculosis* have been extensively analyzed by transcriptional profiling of wild-type and mutant strains in well-characterized experimental models involving uptake of bacteria by macrophages and withdrawal of oxygen or other nutrients (Rustad et al., 2008; Rohde et al., 2012; Betts et al., 2002; Stewart et al., 2002; Deb et al., 2009). Advances in RNA sequencing (RNA-seq) technologies provide an opportunity to extend these studies. In other bacteria, RNA-seq has allowed genome-wide mapping of transcriptional start sites (TSSs) and identification of noncoding RNAs that play an important role in transcriptional and posttranscriptional regulation of gene expression (Wurtzel et al., 2012; Kröger et al., 2012; Perkins et al., 2009; Seo et al., 2012; Sharma et al., 2010; Dugar et al., 2013; Soutourina et al., 2013). RNA-seq has uncovered an extensive landscape of noncoding RNA in *M. tuberculosis* (Arnvig et al., 2011; Arnvig and Young, 2012; Pellin et al., 2012; Miotto et al., 2012) and, in a powerful combination with ChIP-seq, has identified transcriptional units based on binding of RNA polymerase and NusA (Uplekar et al., 2013). The present study extends such analyses by combining genome-wide TSS mapping with total transcriptome sequencing and protein abundance analysis to identify leaderless transcripts and to assess the contribution of leaderless genes to bacterial physiology in a starvation model.

## Results

### Genome-wide Mapping of TSSs

**Figure 1:**
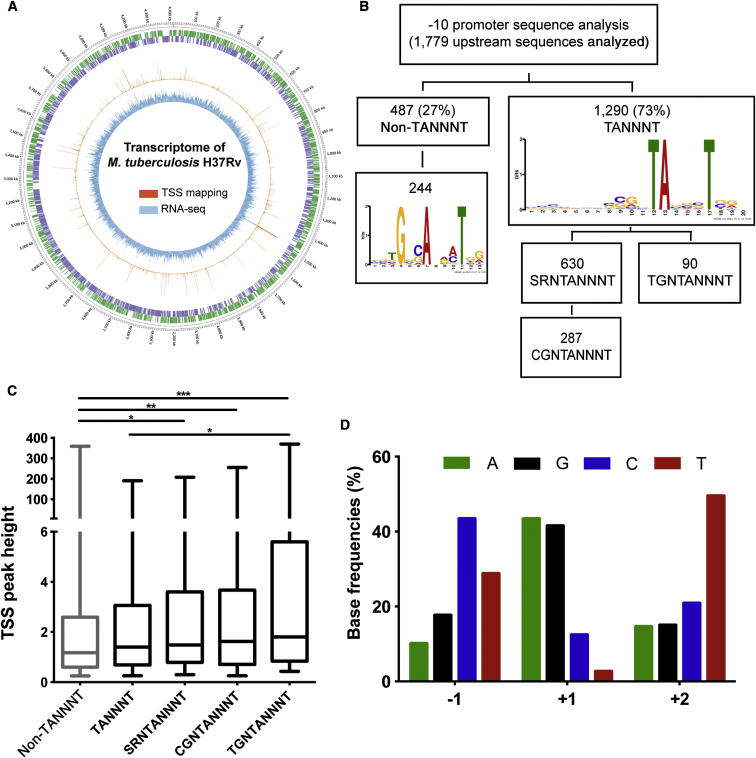
Mapping of TSSs in *M. tuberculosis* (A) Genome visualization shows the transcriptional map of *M. tuberculosis*. Moving from the outer to innermost ring, forward genes are indicated in green and reverse genes in purple; TSS mapping is in orange, and whole-transcriptome expression is in light blue. Circular map was generated using Circos (Krzywinski et al., 2009). (B) The 50 bp upstream sequences of the 1,779 primary TSSs detected were used for motif discovery using MEME. A conserved −10 sequence with consensus TANNNT was found in 73% of the upstream promoter sequences; 7% of these had an extended −10 motif of TGNTANNNT. (C) The extended −10 motif (TGNTANNNT) was associated with maximal promoter activity measured by TSS peak height. Box plots indicate median (horizontal line), interquartile range (box), and minimum and maximum values (whiskers). Statistically significant differences are indicated for p values of ^∗^p < 0.05, ^∗∗^p < 0.01, and ^∗∗∗^p < 0.001. (D) Base preference at the transcription initiation start point is presented. The percent representation of each base is shown for positions −1, +1, and +2 among the 1,779 primary TSSs. See also Figure S1 and Table S1.

Illumina-sequencing technology was applied to sequence the whole transcriptome from three independent biological replicates of exponentially growing *M. tuberculosis* H37Rv (Figure 1A). After removing reads mapping to ribosomal genes (which on average corresponded to 87% of the mapped reads), we obtained a mean 12-fold coverage of the H37Rv genome. Calculation of pairwise correlation coefficients demonstrated a high degree of reproducibility between sequencing runs (Table S1). For the coding transcriptome, the number of reads mapped to each coding sequence (CDS) was calculated and corrected for gene length and library depth to generate normalized reads per kilobase per million mapped reads (RPKM) values (Mortazavi et al., 2008) (Table S1). A total of 2,524 CDSs had RPKM values of five or more, representing 63% of the annotated genome. The average level of antisense transcription within CDS regions was 10.5% of that in the sense orientation.

To obtain single-base resolution of TSSs, we used a selective approach that preferentially enriches for the 5′-triphosphate ends of primary transcripts (Sharma et al., 2010). TSSs were identified by a significant increment in read count compared to average background and quantified based on read count over the subsequent 50 bp. TSSs were confirmed when present in at least two of three replicates (∼80% of total mapped TSSs). For the subset of previously characterized promoters, comparison with published data demonstrated a strong concordance between sequence-based global TSS mapping and results obtained by conventional primer extension or 5′ RACE (Table S2). A total of 4,164 TSSs were identified and annotated according to genome position as previously described by Sharma et al. (2010) (Figure 1A). There were 2,388 primary TSSs associated with upstream regions of annotated coding genes. In those cases when more than one primary TSS was associated with a single CDS, the TSS with maximum peak height was designated as primary, and the others were considered as alternative TSSs. A primary TSS was identified for 1,779 genes, accounting for 44% of the total genome; 338 out of 1,779 (19%) had multiple TSSs. Antisense TSSs were detected for 758 of annotated CDSs (18.9%), 1,064 CDSs (26%) had an internal TSS within the annotated CDS, and 8% of the intergenic regions had a TSS that was not associated with previously identified noncoding RNA. The genome position of all TSSs is recorded in Table S1 along with their categorization as primary, antisense, internal, or secondary (see Figure S1).

### Characterization of Mycobacterial Promoters

Sequences 50 bp upstream of TSSs were used for motif discovery by MEME (Bailey and Elkan, 1994) (Table S1). A conserved TANNNT −10 motif was found centered 7–12 bp upstream of 73% of the primary TSSs (Figure 1B), but no conserved −35 motif was identified. The same −10 motif was found for 50% of internal TSSs and 76% of antisense TSSs. For 49% of the TANNNT promoters, the −10 motif was preceded by the 3 bp consensus [G/C][A/G]N (SRN), with CGN found in 45% of the cases (Figure 1B). Only 7% of conserved TANNNT motifs were preceded by the TGN sequence described as forming an extended −10 consensus associated with enhanced transcriptional activity (Agarwal and Tyagi, 2003). To test whether the extended −10 promoter plays a role in determining promoter strength in mycobacteria, we compared the median peak heights among non-TANNNT, TANNNT, SRNTANNNT, CGNTANNNT, and TGNTANNNT promoters (Figure 1C). The extended −10 motif was found to be associated with a significant increase in promoter activity (Kruskal-Wallis test, p = 0.0107). Among TSSs that did not have a TANNNT motif at the −10 region, the conserved motif shown in Figure 1B was found in 50% of the upstream sequences. TSS mapping confirmed that *M. tuberculosis* can use any base to initiate transcription (Newton-Foot and Gey van Pittius, 2013), although a purine base is preferred in 85% of the cases (A, 43.4%, and G, 41.5%) (Figure 1D). The start site is most commonly flanked by a pyrimidine base; in particular, C and T are the residues most commonly used at positions −1 and +2, respectively.

### High Abundance of Leaderless Transcripts in *M. tuberculosis*

For 505 *M. tuberculosis* genes, the primary TSS was located within 5 bp of the translational start codon, generating a substantial subset of leaderless mRNAs. Among the 2,524 genes expressed during exponential phase, 22% are expressed as leaderless transcripts lacking a 5′ UTR. An additional 47 genes had an additional alternative TSS, providing the option of transcription with or without a 5′ UTR.

Identification of leaderless transcripts is contingent on the accuracy of predictive algorithms used to annotate translational start sites. A recent study proposed the reassignment of more than 600 start codons in *M. tuberculosis* (DeJesus et al., 2013). Mapping to these alternative start codons removed 33 transcripts from the leaderless category and categorized an additional set of 113 genes as leaderless transcripts (Table S3).

For the 1,274 genes showing evidence of a transcribed 5′ UTR, we found 5′ UTR lengths similar to those described in other bacteria, with a median length of 55 nucleotides (Wurtzel et al., 2012; Kröger et al., 2012; Sharma et al., 2010; Irnov et al., 2010) (Figure S1). Transcripts with a 5′ UTR were further categorized by the presence or absence of a Shine-Dalgarno sequence. Shine-Dalgarno sequences are poorly defined in the GC-rich mycobacterial genome (Ma et al., 2002). We compared the results of analysis of the presence of a purine-rich hexamer between 1 and 40 bp upstream of the annotated start codons with two previous Shine-Dalgarno annotations (Zheng et al., 2011; Lew et al., 2011). We identified a core set of 544 genes with a Shine-Dalgarno motif predicted by all three algorithms and a wider set of 1,251 genes predicted by at least two of the three computational approaches. Depending on the stringency of the predictive analysis used to define Shine-Dalgarno sequences, 269 (23%) or 551 (31%) of the genes for which we identified a primary TSS are categorized as having a 5′ UTR Shine-Dalgarno sequence. The remaining 670 genes that were expressed at a level of RPKM of five or more but had no primary TSS were assigned to operons based on alignment and proximity to genes with a primary TSS. Table 1 summarizes the composition of the *M. tuberculosis* transcriptome divided into leaderless, Shine-Dalgarno, or UTR categories on the basis of primary TSS and operon organization (RPKM and TSS data for each gene are recorded along with UTR and operon designation in Table S1).

### Differential Expression of Genes Encoded by Leaderless and Shine-Dalgarno mRNAs

**Figure 2:**
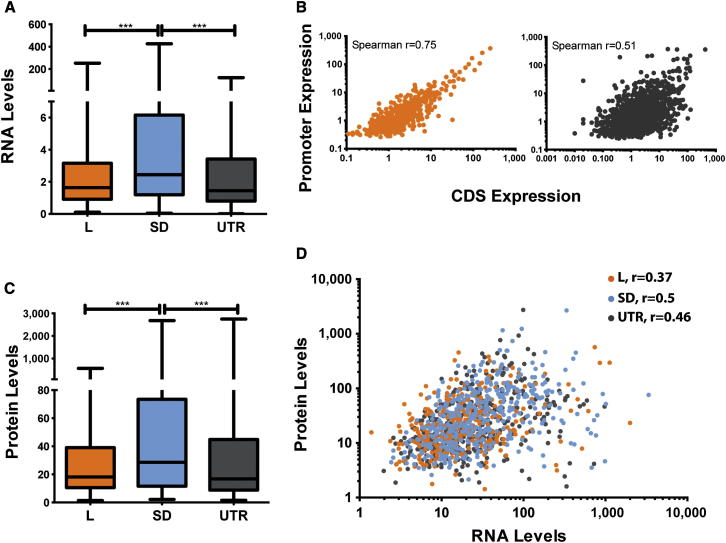
Leaderless Genes Are Expressed at a Lower Level during Exponential Growth (A) and (C) show box plots indicating median (horizontal line), interquartile range (box), and minimum and maximum values (whiskers). Statistically significant differences are indicated (^∗∗∗^p < 0.001). (A) RNA levels (RPKM values) during exponential growth across leaderless (L), Shine-Dalgarno (SD), and UTR genes (identified on the basis of a primary TSS or operon organization) are shown. See also Figure S2 for a similar analysis using more stringent criteria for identification of Shine-Dalgarno genes. (B) Correlation between promoter expression (primary TSS strength) and the associated downstream gene (RPKM values) for leaderless (orange) and leader (gray) genes is shown. (C) Protein levels (measured as ion counts) during exponential growth across leaderless, Shine-Dalgarno, and UTR genes are shown. y Axis represents ion counts rescaled by dividing by 10^6^ in order to reduce axis values. (D) Correlation between protein abundance and mRNA expression is presented. The plot shows an overlay of genes encoded on leaderless (orange), Shine-Dalgarno (blue), and UTR (gray) transcripts. Plots for individual transcript categories are included in Figure S2. See also Figure S2 and Tables S1 and S4.

Comparison of RPKM levels for different transcript categories revealed significantly higher median RNA levels for Shine-Dalgarno genes in exponentially growing cultures (Kruskal-Wallis test, p < 0.0001; Figure 2A). This difference was consistent using different Shine-Dalgarno prediction algorithms (Figure S2) and confirms bioinformatic predictions of an association between Shine-Dalgarno genes and higher levels of expression (Ma et al., 2002). For leaderless transcripts, there was a good correlation between TSS peak height and reads mapping to the associated downstream CDS (Spearman r = 0.75; Figure 2B). In the case of transcripts with a 5′ UTR, the correlation was weaker (Spearman r = 0.51; Figure 2B). The correlation between TSS expression and CDS RPKM increased to 0.6 following removal of genes for which less than 80% of the 5′ UTR is covered by reads. Genes with a poor correlation between TSS expression and RPKM are likely to reflect the presence of attenuated 5′ UTR transcripts (as generated by riboswitches, for example) and cases of incorrect attribution of a 5′ UTR to a downstream gene (rather than as an intergenic sRNA, for example). A recent global analysis of mRNA stability in *M. tuberculosis* concluded that more abundant transcripts have shorter half-lives (Rustad et al., 2013). Consistent with this analysis, mapping of the different transcript categories onto the data of Rustad et al. (2013) revealed a significantly higher median half-life for leaderless as compared to Shine-Dalgarno transcripts (Kruskal-Wallis test, p < 0.0001; Figure S2).

We anticipated that leaderless mRNAs may be translated less efficiently than mRNAs carrying 5′ Shine-Dalgarno sequences. To test this, we carried out parallel shotgun proteomic analysis of extracts from the replicate exponential cultures of *M. tuberculosis* H37Rv used in the transcriptome analysis. We detected signals matching peptides from 1,518 proteins in total; 1,426 proteins were identified in all three replicates (Table S4). We identified protein products for 1,299 (46%) of the 2,825 genes detected at the transcriptional level (i.e., having a primary TSS or RPKM of five or more). The rate of protein detection was similar for leaderless mRNAs (350 out of 722; 48%), transcripts with a 5′ UTR (393 out of 942; 42%), and genes with Shine-Dalgarno transcripts (417 out of 781; 53%). Based on selected ion counts, median protein levels differed between transcript categories, with Shine-Dalgarno genes again being significantly higher (Kruskal-Wallis test, p < 0.0001; Figure 2C). Comparable to previous studies in bacteria and mammals by Schwanhäusser et al. (2011) and Maier et al. (2009), the direct comparison of mRNA and protein levels revealed a weak correlation, with Spearman correlation coefficient (r) in the range of 0.37–0.5 (Figure 2D). A lower correlation was found for the leaderless category, but overlap of the three data sets provided no evidence for an overall difference in the ratio of mRNA:protein abundance for different transcript categories (Figure S2).

### Leaderless Transcripts Are Differentially Distributed among Functional Gene Classes

**Figure 3:**
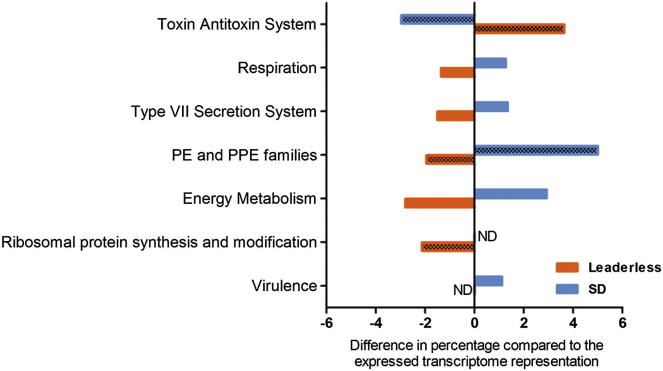
Leaderless and Shine-Dalgarno Transcripts Are Differentially Represented among Different Functional Gene Classes The distribution of genes encoded by leaderless and Shine-Dalgarno transcripts across different functional classes was compared among the expressed transcriptome representation. Values on the x axis represent a difference in percentage; positive values indicate overrepresentation of a particular functional class with respect to whole-transcriptome representation, whereas negative values indicate underrepresentation. All functional categories shown were statistically significant after chi-square test analyses, and patterned bars further denote the functional categories that remained significant after multiple testing correction. ND, no difference. See also Table S1.

The distribution of genes expressed in the form of leaderless transcripts was analyzed with respect to different functional classes of the encoded proteins (Figure 3). Leaderless mRNAs were almost completely absent from the sets of genes involved in respiration and energy metabolism. There were no leaderless mRNAs among the 49 genes encoding members of the PE/PPE protein families expressed at a level of five or more RPKM, and among genes encoding components of type VII secretion systems, only 4 out of 52 were expressed in the form of leaderless transcripts. None of the ribosomal proteins, or related initiation and elongation factors, was encoded as leaderless mRNAs, but 7 of 14 tRNA synthase genes with a primary TSS were leaderless (2 further tRNA synthases had internal TSSs generating leaderless transcripts consistent with revised start codon predictions; DeJesus et al., 2013). In contrast, leaderless transcripts were predominant among gene pairs encoding toxin-antitoxin modules. Of 37 gene pairs with a primary TSS based on start codons annotated in TubercuList, 32 were leaderless; a further 8 genes pairs had an internal TSS consistent with a leaderless transcript based on an alternative ATG or GTG start codon (Table S5). Analysis of Shine-Dalgarno transcripts revealed a largely reciprocal distribution, with significant enrichment in energy metabolism and PE/PPE categories (Figure 3). A similar distribution was observed using the set of Shine-Dalgarno transcripts predicted at both levels of stringency. This pattern is consistent with previous bioinformatic analyses showing Shine-Dalgarno enrichment among genes involved in energy production and protein synthesis (Nakagawa et al., 2010). The differential distribution of leaderless and Shine-Dalgarno mRNAs among functional classes suggests that the different transcript categories may be preferentially associated with different aspects of mycobacterial physiology and metabolism.

### Differential Expression of Leaderless mRNAs in Response to Starvation

Based on their underrepresentation among gene classes involved in functions required for active bacterial growth, we anticipated that the relative abundance of leaderless mRNAs may increase in nonreplicating cultures of *M. tuberculosis*. We tested this hypothesis using a starvation model in which washed exponentially growing cells are incubated in PBS for 24 hr; this has previously been shown to induce a robust transcriptional response involving upregulation of a set of genes together with widespread downregulation of genes involved in aerobic respiration and macromolecule synthesis (Betts et al., 2002). Triplicate samples were analyzed for total RNA and for TSSs as before (Tables S1 and S6). RNA-seq analysis confirmed previous microarray studies with mRNAs for 795 genes decreasing >2-fold and 611 genes increasing >2-fold (Table S5). As previously described, downregulated genes were enriched for functional classes involved in ribosomal proteins and energy metabolism, including the set of genes regulated by the stringent response (Dahl et al., 2003).

**Figure 4:**
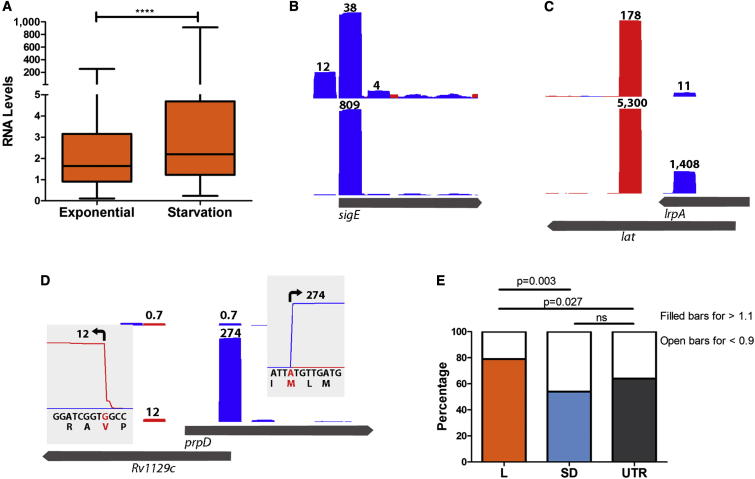
Differential Expression of Leaderless mRNAs in Response to Starvation (A) There was a significant increase in the median level of expression of leaderless mRNAs after 24 hr starvation measured by RPKM for whole genes. (B)–(D) show Artemis traces illustrating TSS mapping for genes strongly upregulated in the starvation model. Bars record the normalized number of mapped reads; the maximum normalized read count is indicated on top of each bar. The position of the TSS corresponds to the left-hand edge of the bar for transcripts in the forward orientation (shown in blue) and to the right-hand edge for transcripts in the reverse orientation (red). In each panel, the top trace is from exponential growth, and the lower trace shows TSS profiles after 24 hr starvation. The genome location is shown at the bottom of each panel. (B) Transcription of *sigE* is driven by three TSSs in exponential phase; only the middle TSS—generating a leaderless mRNA—is upregulated in response to starvation. (C) Upregulation of *lat* (Rv3289c) is accompanied by upregulation of an antisense transcript to *lrpA* (Rv3290c). Closely juxtaposed divergent promoters are a common feature of the *M. tuberculosis* transcriptome. (D) *PrpD* (Rv1130) and its adjacent divergently oriented regulator (Rv1129c) are both upregulated in response to starvation; insets show mapping of TSS to Met 26 and Val 9 start codons, generating leaderless mRNAs. (E) Bar charts indicate the percentage of proteins with abundance ratios (starvation/exponential growth) greater than 1.1 and less than 0.9 for leaderless, Shine-Dalgarno, and UTR categories. ns, not significant. See also Tables S1, S4, S5, and S6.

Although median RNA levels of Shine-Dalgarno genes did not vary significantly in the starved transcriptome compared to exponential growth, there was a significant increase in the median level of leaderless mRNAs in the starved transcriptome (Mann-Whitney U test, p < 0.0001; Figure 4A). Leaderless transcripts were markedly enriched among genes that were most strongly upregulated (Table 2), including members of the σ factor E (*sigE*)/methylcitrate regulatory loop, and the *lrpA/lat* operon (Figures 4B–4D). During starvation, the average fold change increase found for toxin-antitoxin modules was 1.53, with ten gene pairs >2-fold upregulated and one >2-fold downregulated (Table S5). In contrast, leaderless mRNAs encoding tRNA synthases decreased significantly in response to starvation.

In marked contrast to the transcriptional response, the proteome remained largely unchanged in the starved cultures (Table S4). After statistical filtering, the only protein showing >2-fold increase in abundance was Rv0263c, which is expressed as a leaderless transcript. An abundance ratio was calculated for the set of 1,426 proteins measured in both conditions. Contingency analysis showed that the percentage of leaderless encoded proteins with ratios greater than 1.1 (i.e., increased abundance in starvation) was significantly higher than the percentage found for Shine-Dalgarno and UTR-encoded proteins (Figure 4E).

## Discussion

Combination of TSS mapping with total RNA-seq generates a comprehensive overview of the transcriptional landscape of *M. tuberculosis*. Three quarters of the TSSs identified by this approach share the common −10 motif, TANNNT. This has lower stringency than the canonical TATAAT Pribnow box defined for *E. coli* promoters but is similar to results obtained by global TSS mapping in other bacteria (Kröger et al., 2012; Sharma et al., 2010; Yus et al., 2012) and accommodates the consensus sequence previously identified for mycobacterial promoters recognized by the SigA principle σ factor (Newton-Foot and Gey van Pittius, 2013). Although there was no evidence of base preference for the internal NNN region of the −10 sequence, the three residues preceding the motif had a significant influence on promoter activity. Specifically, the presence of TGN, corresponding to an “extended −10 promoter,” was associated with an increase in promoter activity measured by TSS peak height. This is consistent with previous results from single-gene analyses and with enhanced expression of *inhA* associated with a mutation that generates an extended −10 consensus in drug-resistant strains of *M. tuberculosis* (Ramaswamy et al., 2003). We were unable to identify any consensus motifs associated with the −35 region of *M. tuberculosis* promoters, leading us to infer that the −10 sequence is a dominant determinant of RNA polymerase recognition. Consistent with this, from ∼16,400 copies of the TANNNT motif in the genome of *M. tuberculosis*, we detected over 3,100 TANNNT-associated TSSs, and SNPs that create a new TANNNT consensus in clinical isolates often give rise to new TSSs (Rose et al., 2013). There are 511 copies of the TGNTANNNT extended −10 motif in the genome and 90 TGNTANNNT-associated TSSs. The remaining 27% of TSSs that lack a TANNNT consensus may correspond to promoters recognized by alternative σ factors.

TSS mapping can make an important contribution to improved genome annotation. A recent bioinformatic analysis highlighted over 600 *M. tuberculosis* CDSs for which a start codon differing from that used in the current genome annotation could be considered (DeJesus et al., 2013). For 93 of these genes, TSS mapping identified a promoter that fell within the coding region of the originally annotated protein but would be appropriately placed to act as a primary TSS based on the alternative start codon (Table S3). Although protein analysis will be required for definitive identification of translational start sites, experimental data from TSS mapping provide support for the revised bioinformatic predictions for these genes.

A striking feature of the *M. tuberculosis* transcriptome is a high percentage of leaderless transcripts in which the TSS is coincident with the proposed translational start. Although the precise number of leaderless genes is contingent on start codon predictions, around 500 genes—a quarter of all genes with a primary TSS—were identified as leaderless. This compares to a total of only 18, 23, and 83 leaderless mRNAs identified by comparable analysis of the transcriptomes of *E. coli*, *Salmonella typhimurium*, and *Klebsiella pneumonia* (Kröger et al., 2012; Seo et al., 2012; Sharma et al., 2010). TSS mapping provides experimental evidence that supports previous bioinformatic predictions of a high proportion of leaderless mRNAs in selected eubacterial genera, including the Actinobacteria (Zheng et al., 2011). Leaderless genes are not evenly distributed across different functional classes. There is a significant underrepresentation of leaderless mRNAs among genes involved in core growth functions, including energy generation and ribosomal proteins. Leaderless transcripts are also largely absent from mycobacteria-specific gene families encoding type VII secretion systems and PE/PPE proteins but are prominently overrepresented among the abundant class of toxin-antitoxin modules. Transcripts with a 5′ UTR that include a Shine-Dalgarno sequence have a distribution that is largely reciprocal to that of leaderless transcripts.

Consistent with their differential distribution among genes with active growth functions, the median level of reads mapping to leaderless mRNAs was significantly lower than that of reads mapping to Shine-Dalgarno mRNAs during exponential growth. When growth was arrested in a starvation model involving resuspension of *M. tuberculosis* in PBS, there was a significant increase in the relative expression of leaderless mRNAs. Highly upregulated leaderless transcripts included homologs of Kipl-KipA regulators that are linked to sporulation in *Bacillus subtilis* (Jacques et al., 2011) and differentially expressed in *phoP* mutants of *M. tuberculosis*, genes regulated by the “feast to famine” leucine response protein (LrpA, Rv3291c) (Reddy et al., 2008; Deng et al., 2011), and components of a stress-related regulatory circuit involving *sigE* and genes from the methylcitrate cycle (Datta et al., 2011; Ghosh et al., 2011). *SigE* itself can be transcribed from several alternative promoters (Donà et al., 2008), but the >10-fold increase during starvation is driven solely by the leaderless transcript. Genes expressed as leaderless transcripts have a secondary role during exponential growth of *M. tuberculosis* but may play an important role in the physiology of nonreplicating cells.

In *E. coli*, leaderless transcripts are translated with low efficiency (Moll et al., 2002, 2004; Moll and Engelberg-Kulka, 2012), though this can be reversed under stress conditions or as a consequence of changes to the ribosome mediated by treatment with kasugamycin (Kaberdina et al., 2009), or by cleavage of the 3′ region of 16S rRNA by MazF toxin (Vesper et al., 2011). Proteome analysis of exponentially growing *M. tuberculosis* revealed a significantly lower median level of expression for proteins encoded by leaderless transcripts in comparison to those encoded by Shine-Dalgarno transcripts. Quantitative comparison of mRNA and protein abundance revealed a weak correlation with overlapping plots for the two sets of genes. In the absence of information about relative degradation rates, we are unable to determine whether the higher abundance of Shine-Dalgarno proteins is a consequence of translational bias or simply a reflection of different transcript levels. It is of interest in this context that the data set reported by Rustad et al. (2013) shows a longer median half-life for leaderless as compared to Shine-Dalgarno transcripts. In *E. coli*, a shorter half-life is associated with a reduction in translation; it remains to be determined whether this paradigm also holds true for *M. tuberculosis*. In contrast to the dynamic transcriptional response, we observed only minimal changes to the proteome after 24 hr incubation in the starvation model. Interestingly, a recent proteome analysis of culture filtrate proteins prepared after 6 weeks incubation in PBS reported increased abundance of toxin-antitoxins and decreased type VII secretion proteins, a profile that is consistent with a bias toward leaderless transcripts (Albrethsen et al., 2013). Consistent with the *E. coli* model, disruption of the Shine-Dalgarno sequence significantly impairs translation of Shine-Dalgarno genes in *M. tuberculosis* (Woong Park et al., 2011), but it is possible that these bacteria have an alternative pathway for initiation of leaderless translation. There is a strict requirement of an ATG start codon for translation of leaderless mRNAs in *E. coli*, for example in Brock et al. (2008), but the frequency of ATG and GTG start codons is similar between leaderless and UTR genes in *M. tuberculosis*. Translation of leaderless mRNAs can be initiated on 70S ribosomes in *E. coli*, and accumulation of 70S ribosomes under stress conditions (Trauner et al., 2012) may influence translation efficiency in *M. tuberculosis*.

In summary, we have shown that more than a quarter of the total transcriptome in *M. tuberculosis* is expressed in the form of leaderless mRNAs. Leaderless transcripts are differentially distributed according to functional class, with a low frequency among genes involved in active replication and a higher frequency among genes expressed in nondividing cells. An understanding of the transcription and translation of leaderless mRNAs may provide insights into persistent infection with *M. tuberculosis* and uncover strategies for drug discovery.

## Experimental Procedures

Detailed descriptions are given in the Supplemental Experimental Procedures.

### Culture Conditions and RNA Isolation

*M*. *tuberculosis* H37Rv (SysteMTb) was grown in Middlebrook 7H9 medium supplemented with 0.4% glycerol, 0.085% NaCl, 0.5% BSA, and 0.05% Tyloxapol in roller bottle culture (2 rpm at 37°C). For starvation experiments, exponentially growing bacteria were washed, resuspended in PBS supplemented with 0.025% Tyloxapol, and maintained in roller bottle culture for a further 24 hr (Gengenbacher et al., 2010). RNA was isolated from triplicate PBS-washed cultures as previously described (Arnvig et al., 2011). RNA was treated with TURBO DNase (Ambion) until DNA free. The quality of RNA was assessed using a NanoDrop (ND-1000; Labtech) and Agilent bioanalyzer.

### Construction of cDNA Libraries for Illumina Sequencing

RNA samples from triplicate exponential and starved cultures were used to construct cDNA libraries for whole-transcriptome and TSS mapping by vertis Biotechnologie AG (http://www.vertis-biotech.com/). The 12 obtained cDNA libraries were multiplexed and sequenced as single-end reads on a single lane on the Illumina HiSeq 2000 sequencing machine by vertis Biotechnologie AG.

### Read Mapping and Profile Generation

Quality of the Illumina-produced fastq files was assessed, and good quality reads were mapped to the reference sequence of *M. tuberculosis* H37Rv (GenBank AL123456) as single-end data with BWA (Li and Durbin, 2009). Genome coverage was calculated using BEDTools (Quinlan and Hall, 2010). RPKM values were calculated (Mortazavi et al., 2008). For TSS calling, custom perl scripts were written to calculate the increment in reads from one genome position to the consecutive base across the genome and all genomic positions where an increment significantly above the average background was detected were extracted as candidate TSSs. The TSS peak height was considered as representative of the level of expression of the TSS. True TSSs were considered when a given genome position was called in at least two out of the three biological replicates allowing ± 10 bp tolerance.

### TSS Annotation

To build a genome-wide TSS map for *M. tuberculosis*, custom perl scripts were used for the automated annotation of the putative TSSs detected according to genomic distribution similarly as previously described by Sharma et al. (2010) (Figure S1). TubercuList annotation (Release R25, April 2012) was used as the annotation reference of the *M. tuberculosis* genome (Lew et al., 2011). A “primary TSS” was defined when a TSS was detected within a distance ≤500 bp upstream of annotated ORFs. “Secondary TSSs” were assigned to TSSs located on intergenic regions and separated more than 500 bp from the adjacent annotated ORFs. TSSs situated inside of an annotated CDS on the opposite strand were classified as “antisense TSSs,” and “internal TSSs” were defined when the TSS was inside of an annotated CDS on the same strand. When more than one “primary” TSS was associated with the same ORF, TSS peak height was used to discriminate between the primary TSS (corresponding to the strongest TSS according to peak height value) and “alternative primary” TSSs.

### Classification of *M. tuberculosis* Genes with a Primary TSS

*M. tuberculosis* genes with a primary TSS detected were classified into three main categories according to their 5′ UTR length and translation initiation signal. Genes with a 5′ UTR between −5 and +5 bp were classified as “leaderless.” The remaining genes with a primary TSS and UTRs longer than 5 bp were classified according to the presence/absence of a Shine-Dalgarno sequence for translation initiation. A total of 1,414 genes were predicted as having a Shine-Dalgarno sequence upstream of the translation initiation site. The Shine-Dalgarno-predicted genes were compared with the 1,184 Shine-Dalgarno genes from TubercuList and the 1,365 Shine-Dalgarno-predicted genes by Zheng et al. (2011). The set of 1,251 genes shared by at least two of the predictions was considered a Shine-Dalgarno-like representative. The subset of genes with a 5′ UTR and a Shine-Dalgarno-like signal predicted was classified as a Shine-Dalgarno-like representative. Finally, the remaining genes where a Shine-Dalgarno-like signal was not detected were classified as UTR. The remaining genes for which a primary TSS was not detected but that were expressed at the whole transcriptome level were assigned to operons based on alignment and proximity to genes with a primary TSS.

### Genome-wide Proteomics

Bacterial cell pellets were dissolved in lysis buffer and disrupted by applying two 40 s cycles with FastPrep-24 (MP Biomedicals). Protein concentration was determined using a BCA assay according to the manufacturer’s protocol (Thermo Fisher Scientific). Proteins were reduced and alkylated, followed by a tryptic digest. The peptide solution was desalted by C18 reversed-phase columns, dried under vacuum, and resolubilized to a final concentration of 1 mg/ml.

One microgram of each peptide sample was analyzed on an LTQ Orbitrap XL mass spectrometer (Thermo Fisher Scientific). The acquired MS2 spectra were searched with OMSSA, XTandem, and MyriMatch against an *M. tuberculosis* H37Rv protein database (TubercuList v.2.3, April 2011) additionally containing reversed sequences of all proteins in the database. Only peptides at a false discovery rate (FDR) of less than 1% were taken into consideration for further analysis. For MS1-based label-free quantification, the openMS v.1.8 framework was used (Weisser et al., 2013). Signals were normalized on peptide feature level such that the median signal in each sample is the same. Abundances of the three most intense peptides were averaged to get a protein abundance value. The same peptides were used for protein quantification across all samples, and proteins with less than three peptides were quantified as well.

### Statistical Analysis

For functional enrichment analysis, GraphPad Prism v.5.03c was used to compare the frequencies of different functional categories in respect to the H37Rv-expressed transcriptome using two-tailed chi-square tests. When multiple chi-square tests were performed, multiple testing correction was applied using the FDR method implemented in R. Nonparametric tests (Kruskal-Wallis or Mann-Whitney U tests) were used to evaluate differences among median levels of expression. Protein quantification values were rescaled by dividing by 10^6^. mRNA-protein correlations were determined using the Spearman rank coefficient.

### Differential Expression Analyses

For whole-transcriptome differential expression calling, genome coverage of reads mapping to genes was used for statistical testing using DESeq (Anders and Huber, 2010) implemented in the R statistical environment. Differentially expressed genes were considered when fold changes between exponential growth and starvation were greater than or equal to 2-fold and the corresponding adjusted p value was less than 0.01. For differential expression analysis of TSSs, the maximum number of reads mapped within a 50 bp range from the TSS (peak height) was used for DESeq analysis.

## Author Contributions

T.C., K.B.A., and D.B.Y. designed the research. T.C. and O.T.S. performed the experiments. T.C., O.T.S., I.C., and D.B.Y. analyzed the data. T.C. and G.R. performed bioinformatic analysis. T.C., O.T.S., G.R., I.C., K.B.A., R.A., and D.B.Y. wrote the paper.

## Figures and Tables

**Table 1:** Categorization of *M. tuberculosis* Transcripts on the Basis of TSS Mapping

	H37Rv Genes with a Primary TSS	H37Rv Genes Assigned to Operons
Shine-Dalgarno	551	231
UTR	676	266
Leaderless	505	162
Alternative leaderless	47	8

**Table 2:** Genes Upregulated >10-Fold after 24 hr in the Starvation Model

Gene	Symbol	Exponential Mean Reads	Starvation Mean Reads	Fold Change	Adjusted p Value	TSS Genome Location	Operon	Start Codon	Category
Rv1131^a^	prpC	2.4	586.2	247.62	4.82 × 10^−70^	1254630	Rv1130		L op
Rv1130^a^	prpD	5.6	1,359.6	244.54	1.24 × 10^−63^	1254630		Met 26^c^	L
Rv3289c^b^	Rv3289c	37.8	2,004.9	52.97	3.40 × 10^−274^	3671794	Rv3290c		L op
Rv2662	Rv2662	4.3	141.0	33.10	4.89 × 10^−59^	2980909			
Rv0264c	Rv0264c	10.6	316.0	29.75	8.70 × 10^−104^	316436		Met −7^d^	L
Rv3290c^b^	lat	477.7	1,3412.2	28.07	1.98 × 10^−289^	3671794			L
Rv0263c	Rv0263c	23.4	624.5	26.72	1.37 × 10^−147^	316436	Rv0264c		L op
Rv0260c	Rv0260c	0.8	19.7	24.50	4.00 × 10^−10^	312659			L^e^
Rv1057	Rv1057	13.0	227.5	17.53	6.29 × 10^−18^	1179215			SD
Rv2034	Rv2034	11.5	200.2	17.48	1.47 × 10^−53^	2281292			L
Rv1129c^a^	Rv1129c	8.5	142.1	16.80	1.13 × 10^−30^	1254510		Val 9^d^	L
Rv0188	Rv0188	87.5	1,319.3	15.08	2.08 × 10^−146^	219429			SD
Rv1371	Rv1371	1.6	23.7	15.03	2.44 × 10^−10^	1544131^f^			
Rv0789c	Rv0789c	25.4	372.1	14.64	9.59 × 10^−90^	883924		Met 43^d^	L
Rv3447c	eccC4	1.6	22.4	14.04	1.07 × 10^−09^	ND			
Rv0516c	Rv0516c	231.4	3,156.9	13.64	1.94 × 10^−176^	608588			SD
Rv3354	Rv3354	31.5	426.2	13.54	2.89 × 10^−50^	3769111			L
Rv1221^a^	sigE	242.3	3,071.1	12.67	1.57 × 10^−167^	1364412			L
Rv3353c	Rv3353c	2.4	29.8	12.44	3.80 × 10^−12^	3769000			L^e^
Rv0467^a^	icl1	126.5	1,525.1	12.06	4.49 × 10^−21^	557436			SD
Rv3310	sapM	20.4	243.0	11.90	5.27 × 10^−34^	3697198			L^g^
Rv1809	PPE33	42.8	507.0	11.84	3.67 × 10^−19^	2047593	Rv1806		SD op
Rv3288c^b^	usfY	56.0	650.4	11.61	8.88 × 10^−49^	3671794	Rv3290c		L op
Rv1555	frdD	3.1	34.6	10.98	5.75 × 10^−13^	1757400	Rv1552		U op
Rv1542c	glbN	1.8	20.0	10.83	5.88 × 10^−08^	1744836			L
Rv1554	frdC	2.6	28.5	10.76	2.25 × 10^−06^	1757400	Rv1552		U op
Rv2323c	Rv2323c	24.1	258.4	10.74	1.61 × 10^−62^	2596209		Met 21	L^h^
Rv2699c	Rv2699c	16.6	173.0	10.42	3.25 × 10^−34^	3015010			SD

Categories of highly expressed leaderless transcripts are highlighted by ^a^ and ^b^. ND, TSS not detected. SD, Shine-Dalgarno.

a *sigE*/methylcitrate network.

b *LrpA/lat* regulon.

c Start codon based on *Mycobacterium marinum*, Corynebacteria, and Streptomyces (DeJesus et al., 2013 propose Met22) (Figure 4D) is shown.

d Start codon as proposed by DeJesus et al. (2013) is presented.

e Leaderless TSS falls below cutoff for H37Rv but is clearly seen in clinical isolates (Rose et al., 2013).

f Gene is composed of two functional domains (divided by frameshift in some strains); internal TSS upstream of desaturase domain is shown.

g TubercuList annotation generates leaderless mRNA; DeJesus et al. (2013) propose Leu 19 start codon.

h Start codon is based on *M. marinum* and *Bacillus*; DeJesus et al. (2013) propose Met46.

## Accession Numbers

The EBI Array Express accession number for the RNA-seq data reported in this paper is E-MTAB-1616. The mass spectrometry proteomics data have been deposited in the ProteomeXchange Consortium (http://proteomecentral.proteomexchange.org/) via the PRIDE partner repository (Vizcaíno et al., 2013) under accession number PXD000483.

## Supplemental Information

Document S1. Supplemental Experimental Procedures, Figures S1 and S2, and Table S2

Table S1. Summary of Sequencing Runs and RNA-Seq and TSS Mapping during Exponential Growth, Related to Figures 1, 2, 4A, and Results Sheet 1A is a summary of sequencing runs and correlations between replicate samples. Sheet 1B is a listing of sense and antisense reads and RPKM values for all CDS during exponential growth. Sheet 1C lists all primary TSSs mapped during exponential growth. Sheet 1D lists all antisense TSSs mapped during exponential growth. Sheet 1E lists all internal TSSs mapped during exponential growth. Sheet 1F lists all the TSSs mapped during exponential growth and their genomic location.

Table S3. TSS Mapping and Reannotation of Start Codons, Related to Results Excel file showing the mapping of alternative translational start codons proposed by (DeJesus et al., 2013) to TSS data.

Table S4. Protein Abundance for the 1,518 Proteins Detected, Related to Figures 2C, 2D, 4E, and Results Excel file listing protein abundance (ion counts) for the 1,518 proteins detected in this analysis.

Table S5. Differential Expression during Starvation, Related to Figure 4 and Results Sheet 5A is a list of the CDS differentially expressed during starvation. Sheet 5B is a list of the differentially expressed primary TSSs during starvation and sheet 5C lists toxin-antitoxin modules and their differential expression during starvation.

Table S6. RNA-Seq and TSS Mapping during Starvation, Related to Figure 4 Sheet 6A is a listing of sense and antisense reads and RPKM values for all CDS during starvation. Sheet 6B lists all primary TSSs mapped during starvation. Sheet 6C lists all antisense TSSs mapped during starvation. Sheet 6D lists all internal TSSs mapped during starvation. Sheet 6E lists all the TSSs mapped during starvation and their genomic location.

Document S2. Article plus Supplemental Information
