# Genome-wide Mapping of Transcriptional Start Sites Defines an Extensive Leaderless Transcriptome in *Mycobacterium tuberculosis*

**DOI:** 10.1016/j.celrep.2014.01.004

**Published:** 2014-01-30

**Authors:** Teresa Cortes, Olga T. Schubert, Graham Rose, Kristine B. Arnvig, Iñaki Comas, Ruedi Aebersold, Douglas B. Young

(Cell Reports *5*, 1121–1131; November 21, 2013)

In the originally published version of this article, the data in Table S3 were incorrectly sorted. The article has been updated with a new version of the table, and the data have been sorted correctly. The authors regret this error and any confusion that may have resulted.

