# Supplementary material for: Genome-wide Mapping of Transcriptional Start Sites Defines an Extensive Leaderless Transcriptome in Mycobacterium tuberculosis
Source: Cell Rep. 2013 Nov 21;5(4):1121–31. doi: 10.1016/j.celrep.2013.10.031 (PMC3898074; doi:10.1016/j.celrep.2013.10.031)
Supplement: Document S1. Supplemental Experimental Procedures, Figures S1 and S2, and Table S2 [file mmc1.pdf]

Genome-wide Mapping of Transcriptional Start Sites Defines an Extensive Leaderless Transcriptome in *Mycobacterium tuberculosis*

Teresa Cortes, Olga T. Schubert, Graham Rose, Kristine B. Arnvig, Iñaki Comas, Ruedi Aebersold and Douglas B. Young

Supplemental information includes 2 figures, 6 tables and Supplemental Experimental Procedures and references.

Figure S1. Classification scheme for TSSs mapping and 5'UTR lengths distribution. A. Classification scheme for TSSs: primary (P), internal (I), antisense (A) and secondary (S). B. 5' UTR lengths. Distribution and frequency of the length of the 5' UTR of mRNAs (5bp bins) with a primary TSS detected during exponential growth. Red bar indicates the high proportion of genes with UTRs less than 5bp, assigned as leaderless transcripts, in the *M. tuberculosis* transcriptome.

A

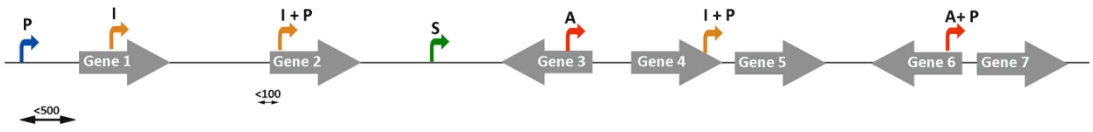

B

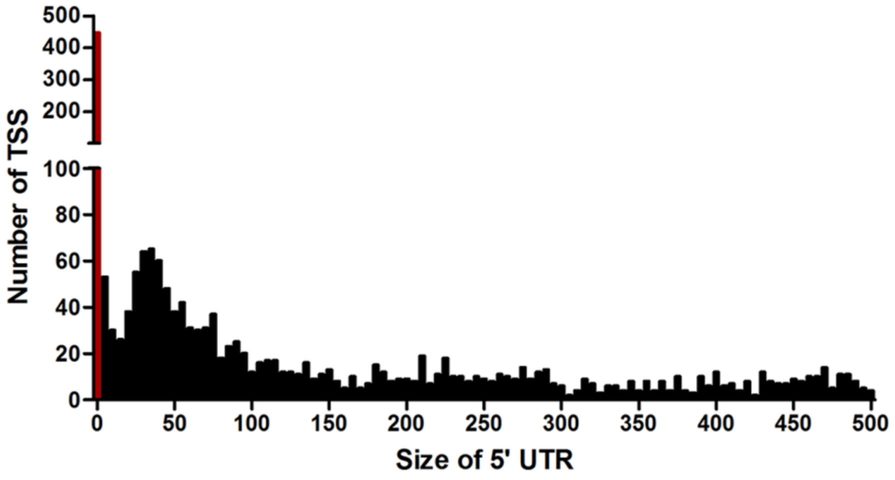

Related to Figure 1A, Experimental Procedures and results sections: "Genome-wide mapping of transcriptional start sites" and "High abundance of leaderless transcripts in *M. tuberculosis*".

Figure S2. mRNA and protein expression among gene categories. A. Parallel analysis to Figure 2A – showing effect of using different Shine-Dalgarno (SD) prediction criteria. Box plots indicating median (horizontal line), interquartile range (box) and minimum and maximum values (whiskers) of RNA levels (RPKM values) during exponential growth across the different subsets of predicted Shine-Dalgarno compared to leaderless. B. Box-plots of half-lives for leaderless (L), Shine-Dalgarno (SD) and UTR (U) mRNAs during exponential growth; ***p < 0.001 (data from Rustad et al., 2012). C. Correlation between protein abundance and mRNA expression for leaderless, Shine-Dalgarno and UTR categories.

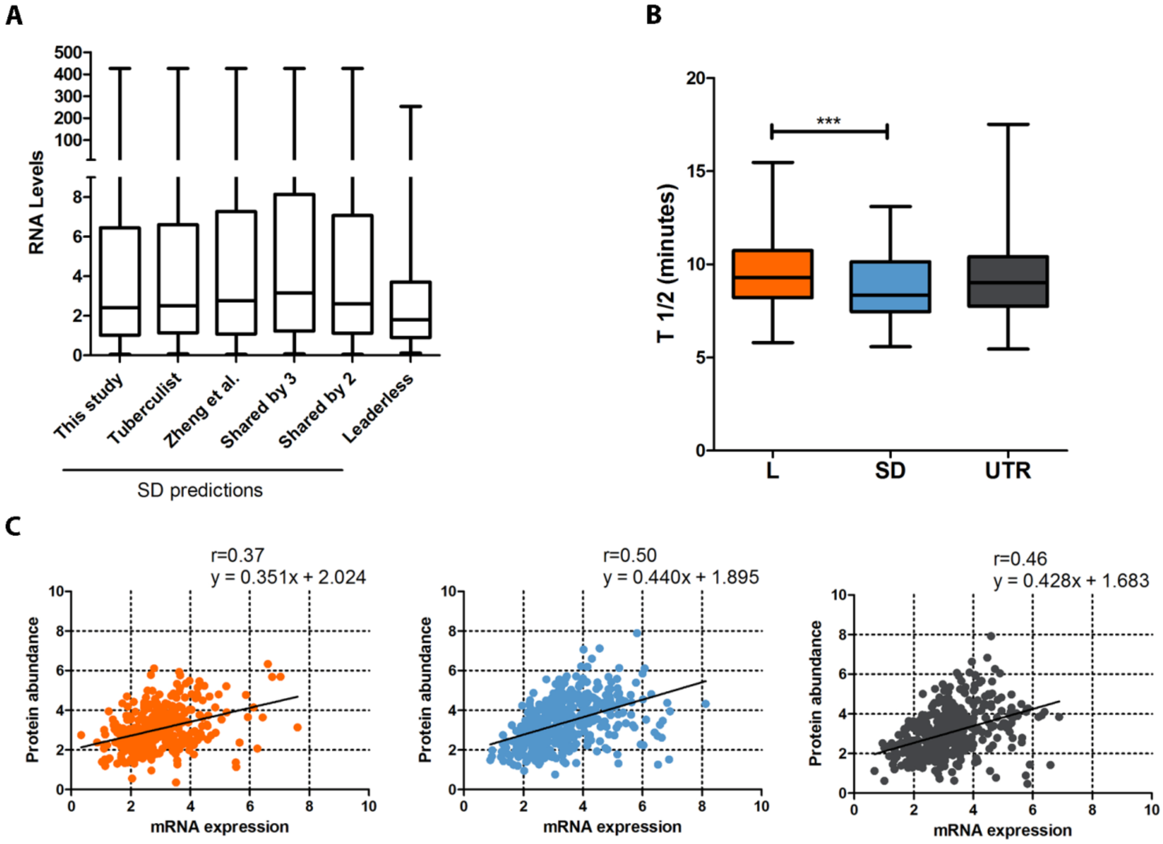

Related to Figure 2A and 2D and results and discussion sections.

Table S1. Summary of sequencing runs and RNA-seq and TSS mapping during exponential growth.

Related to Figures 1, 2 and 4A and results sections: “Genome-wide mapping of transcriptional start sites”, “Characterisation of mycobacterial promoters”, “High abundance of leaderless transcripts in *M. tuberculosis*” and “Differential expression of genes encoded by leaderless and Shine-Dalgarno mRNAs”.

Table S2. TSS match with published single gene data. Related to results section: “Genome-wide mapping of transcriptional start sites”.

Gene	Synonym	Genomic position (experimental, see ref)	TSS mapping Exponential	TSS mapping Starvation	Distance	Reference
Rv0166	fadD5	194927	194917	194917	10	(1)
Rv0282	Rv0282	341976	341976	341980	0	(2)
Rv0341	iniB	409308	409309	409309	-1	(3)
Rv0677c	mmpS5	778966	778967	778967	-1	(4)
Rv0678	Rv0678	778990	778988	778988	2	(4)
Rv0780	purC	873343	873343	873343	0	(5)
Rv0941c	Rv0941c	1052606	1052607	1052607	-1	(6)
Rv1000c	Rv1000c	1117127	1117127	1117127	0	(7)
Rv1054	Rv1054	1176264	1176266	1176266	-2	(8)
Rv1221	sigE	1364358	1364356	1364356	2	(9)
Rv1221	sigE	1364413	1364413	1364412	0	(9)
Rv1284	canA	1437272	1437271	1437271	1	(6)
Rv1284	canA	1437261	1437271		-10	(6)
Rv1528c	papA4	1729469	1729494	1729492	-25	(10)
Rv1737c	narK2	1965428	1965427	1965427	1	(11)
Rv1738	Rv1738	1965575	1965576	1965573	-1	(11)
Rv1812c	Rv1812c	2056330	2056342	2056342	-12	(12)
Rv1813c	Rv1813c	2056330	2056342		-12	(12)
Rv1818c	PE_PGRS33	2062749	2062749	2062749	0	(13)
Rv2059	Rv2059	2315140	2315155	2315155	-15	(2)
Rv2069	sigC	2326827	2326827	2326827	0	(14)
Rv2069	sigC	2326944	2326941	2326941	3	(14)
Rv2150c	FtsZ	2409626	2409625	2409625	1	(15)
Rv2221c	glnE	2492353	2492347		6	(16)
Rv2416c	eis	2715365	2715367		-2	(17)
Rv2583c	rel/spoT	2910216	2910212	2910212	4	(18)
Rv2594c	ruvC	2925416	2925414	2925414	2	(19)
Rv2594c	ruvC	2925479	2925476	2925476	3	(19)
Rv2710	sigB	3022433	3022433	3022431	0	(10)
Rv3102c	ftsE	3471385	3471385	3471375	0	(20)
Rv3130c	tgs1	3496408	3496410	3496410	-2	(21)
Rv3131	Rv3131	3496514	3496512	3496512	2	(21)
Rv3134c	Rv3134c	3500789	3500789	3500790	0	(11)
Rv3219	whiB1	3595603	3595603	3595602	0	(22)
Rv3301c	phoY1	3687630	3687630	3687630	0	(6)
Rv3418c	groES	3837459	3837458	3837458	1	(23)
Rv3616c	espA	4056442	4056443	4056443	-1	(24)
Rv1221	sigE	1364475		1364476	-1	(9)
Rv2358	smtB	2641198		2641196	2	(25)
Rv2736c	recX	3049086		3049086	0	(26)
Rv3804c	fbpA	4266721		4266718	3	(27)

1. Joon, M., Bhatia, S., Pasricha, R., Bose, M., Brahmachari, V. (2010). BMC Microbiol *10,* 128.
2. Maciàg, A., Dainese, E., Rodriguez, G.M., Milano, A., Provvedi, R., et al. (2007). J Bacteriol *189,* 730-740.
3. Allan, D., Steyn, A.J., Weisbrod, T., Aldrich, K., Jacobs, W.R. (2000). J Bacteriol *182*, 1802-1811.
4. Milano, A., Pasca, M.R., Provvedi, R., Lucarelli, A.P., Manina, G., et al. (2009). Tuberculosis *89,* 84-90.
5. Jackson, M., Berthet, F.X., Otal, I., Rauzier, J., Martin, C., et al. (1996). Microbiol *142*, 2439-2447.
6. Hartkoorn, R.C., Sala, C., Uplekar, S., Busso, P., Rougemont, J. et al. (2012). J Bacteriol *194*, 2001-2009.
7. Smollett, K.L., Smith, K.M., Kahramanoglou, C., Arnvig, K.B., Buxton, R.S., et al. (2012), J Biol Chem *287*, 22004-22014.
8. Homerova, D., Surdova, K., Mikusova, K., Kormanec, J. (2007). Arch Microbiol *187,* 185-197.
9. Doná, V., Rodrigue, S., Dainese, E., Palu, G., Gaudreau, L., et al. (2008). J Bacteriol *190,* 5963-5971.
10. Manganelli, R., Voskuil, M.I., Schoolnik, G.K., Dubnau, E., Gomez, M., et al. (2002). *Mol Microbiol 45*:, 365-374.
11. Chauhan, S., Tyagi, J.S. (2008). J Bacteriol *190*, 4301-4312.
12. Bretl, D.J., He, H., Demetriadou, C., White, M.J., Penoske, R.M., et al. (2012). *Infect Immun* 80(9), 3018-3033.
13. Vallecillo, A.J., Espitia, C. (2009). Microb Pathogenesis *46,* 119-127.
14. Chang, A., Smollett, K.L., Gopaul, K.K., Chan, B.H.Y., Davis, E.O. (2012). Tuberculosis *92,* 48-55.
15. Kiran, M., Maloney, E., Lofton, H., Chauhan, A., Jensen, R., et al. (2009). Tuberculosis *89(1),* S60-S64.
16. Hotter, G.S., Mouat, P., Collins, D.M. (2008). Tuberculosis *88*, 382-389.
17. Roberts, E.A., Clark, A., McBeth, S., Friedman, R.L. (2004). J Bacteriol *186*, 5410-5417.
18. Jain, V., Sujatha, S., Ojha, A.K., Chatterji, D. (2005). Gene *351*, 149-157.
19. Dawson, L.F., Dillury, J., Davis, E.O. (2010). J Bacteriol *192*, 599-603.
20. Roy, S., Vijay, S., Arumugam, M., Anand, D., Mir, M., et al. (2011). Curr Microbiol *62*, 1581-1589.
21. Chauhan, S., Tyagi, J.S. (2009). J Bacteriol *191*, 6075-6081.
22. Agarwal, N., Raghunand, T.R., Bishai, W.R. (2006). Microbiology *152*, 2749-2756.
23. Aravindhan, V., Christy, A.J., Roy, S., Ajitkumar, P., Narayanan, P.R., et al. (2009). FEMS Microbiol Lett *292*, 42-49.
24. Hunt, D.M., Sweeney, N.P., Mori, L., Whalan, R.H., Comas, I., et al. (2012). J Bacteriol *194*, 2307-2320.
25. Canneva, F., Branzoni, M., Riccardi, G., Provvedi, R., Milano, A. (2005). J Bacteriol *187*, 5837-5840.
26. Forse, L.N., Houghton, J., Davis, E.O. (2011). Tuberculosis *91*, 127-135.
27. Kremer, L., Baulard, A., Estaquier, J., Content, J., Capron, A., et al. (1995). J Bacteriol *177*, 642-653.

Table S3. TSS mapping and re-annotation of start codons.

Legend:
* Based on DeJesus et al., 2013

Related to results sections: “High abundance of leaderless transcripts in *M. tuberculosis*” and discussion

Table S4. Protein abundance for the 1,518 proteins detected.

Related to Figure 2C, 2D and 4E and to results sections: "Differential expression of genes encoded by leaderless and Shine-Dalgarno mRNAs" and "Differential expression of leaderless mRNAs in response to starvation".

Table S5. Differential expression during starvation.

Related to results section: “Differential expression of leaderless mRNAs in response to starvation” and Figure 4.

Table S6. RNA-seq and TSS mapping during starvation.

Related to results section: “Differential expression of leaderless mRNAs in response to starvation” and Figure 4.

Supplemental Experimental Procedures

Culture conditions and RNA isolation

Mycobacterium tuberculosis H37Rv (SysteMTb) was grown in Middlebrook 7H9 medium supplemented with 0.4% glycerol, 0.085% NaCl, 0.5% BSA and 0.05% Tyloxapol in roller bottle culture (2 rpm at 37°C). For starvation experiments, exponentially growing bacteria were washed, resuspended in PBS supplemented with 0.025% Tyloxapol, and maintained in roller bottle culture for a further 24 hours (Gegenbacher et al., 2010). RNA was isolated from triplicate PBS-washed cultures as previously described (Arnvig et al., 2011). RNA was treated with Turbo DNase (Ambion) until DNA free. The quality of RNA was assessed using a Nanodrop (ND-1000, Labtech) and Agilent bioanalyser.

Construction of cDNA libraries for Illumina sequencing

RNA samples from triplicate exponential and starved cultures were used to construct cDNA libraries for whole transcriptome and TSS mapping by vertis Biotechnologie AG (http://www.vertis-biotech.com). For the synthesis of whole transcriptome cDNA, RNA was fragmented with ultrasound (4 pulses of 30 s at 4°C), then treated with Antarctic phosphatase and re-phosphorylated with polynucleotide kinase (PNK). Fragmented RNA was then poly(A)-tailed using poly(A) polymerase and a RNA adapter was ligated to the 5'-phosphate of the RNA. First-strand cDNA synthesis was performed using an oligo(dT)-adapter primer and M-MLV reverse transcriptase. The resulting cDNA was PCR-amplified to about 20-30 ng/µl using a high fidelity DNA polymerase. For TSS mapping, RNA was enriched for primary transcripts by incubating fragmented, PNK-treated samples with Terminator exonuclease (TEX, Epicentre) which specifically degrades RNA species carrying a 5' monophosphate (5'P). Exonuclease-resistant RNA species (primary transcripts with 5'PPP) were poly(A)-tailed using poly(A) polymerase, followed by treatment with tobacco acid pyrophosphatase (TAP, Epicentre) to degrade 5'PPP to 5'P RNA. cDNA synthesis was carried out as above. The 12 obtained cDNA libraries were multiplexed and sequenced as single-end reads on a single lane on the Illumina HiSeq 2000 sequencing machine by vertis Biotechnologie AG.

Read mapping and profile generation

Quality of the Illumina produced fastq files was assessed and good quality reads were mapped to the reference sequence of *M. tuberculosis* H37Rv [GenBank: AL123456] as single end data using BWA (Li and Durbin, 2009). Trimming of bad quality reads was only performed for whole transcriptome reads. Genome coverage, defined as number of reads mapped per base of H37Rv genome, was calculated using BEDTools (Quinlan and Hall, 2010). RPKM values (reads per kilobase per million mapped reads) were calculated using only sequence reads that mapped to annotated features unambiguously and on the correct strand. For defining gene expression, the threshold of RPKM ≥ 5 was estimated after calculating the 75th percentile of reads mapped to intergenic regions and the associated hypothetical RPKM value considering an average gene length of 1000 bp (Lew et al., 2011). For TSSs calling, custom perl scripts were written to calculate the increment in reads from one genome position to the consecutive base across the genome and all genomic positions where an increment significantly above the average background was detected were extracted as candidate transcription start sites. The TSS peak height was considered as representative of the level of expression of the TSS. TSS peak height was calculated using a custom perl script where the number of reads mapped to the following 50bp downstream of a TSS were considered and the greater value of mapped reads within this range was considered as the peak height for a given TSS. Peak height values were normalized across TSSs. True TSSs were considered when a given genome position was called in at least two out of the three biological replicates allowing ± 10bp tolerance.

Transcription start site annotation

To build a genome-wide TSS map for *M. tuberculosis*, custom perl scripts were used for the automated annotation of the putative transcription start sites detected according to genomic distribution similarly as previously described (Sharma et al., 2010) (Figure S1). TubercuList annotation (Release R25, April 2012) was used as the annotation reference of the *M. tuberculosis* genome (Lew et al., 2011). A *primary TSS* was defined when a TSS was detected within a distance

≤500bp upstream of annotated ORFs; *secondary TSSs* were assigned to TSSs located on intergenic regions and separated more than 500bp from the adjacent annotated ORFs; TSS situated inside of an annotated CDS on the opposite strand were classified as *antisense TSSs*, and *internal TSSs* were defined when the TSS was inside of an annotated CDS on the same strand. When an *internal TSS* was situated either i) less than 5bp from the start of the annotated ORF or ii) inside of an annotated ORF but less than 500bp from the start of the downstream annotated ORF, it was considered as *internal and primary*. Similarly, those antisense TSSs where the downstream gene was in the correct orientation and less than 500bp, were annotated as *antisense and primary*. When more than one *primary* TSSs was associated to the same ORF, TSS peak height was used to discriminate between the *primary* TSS (corresponding to the strongest TSS according to peak height value) and *alternative primary* TSSs.

Classification of *M. tuberculosis* genes with a primary TSS

M. tuberculosis genes with a primary TSS detected were classified into 3 main categories according to their 5' UTR length and translation initiation signal. Genes with a 5' UTR between -5 and +5bp were classified as *leaderless.* The remaining genes with a primary TSS and UTRs longer than 5bp were classified according to the presence/absence of a Shine-Dalgarno (SD) sequence for translation initiation. For Shine-Dalgarno-like signals prediction, we extracted the -40bp upstream regions of the *M. tuberculosis* annotated CDSs and screened for G(A/T)(A/T)AGGAGGT(G/A)ATC as a common reference sequence (Noguchi et al., 2008). We defined nine hexamers derived from the previous sequence and an exact match or one-base mismatch sequence of the motifs was sought using fuzznucc (Rice et al., 2000). As distance of the Shine-Dalgarno sequence to translation initiation site (TIS) is one of the requirements for optimal translation initiation (Kempsell et al., 1992), only hexamers found within 6 to 14 bp from TIS were considered. 1,414 genes were predicted as having a SD sequence upstream of the TIS. The Shine-Dalgarno-predicted genes were compared with the 1,184 Shine-Dalgarno genes from TubercuList and the 1,365 Shine-Dalgarno-predicted genes by (Zheng et al., 2011). The set of 1,251 genes shared by at least two of the predictions were

considered as Shine-Dalgarno-like representatives. After this analysis, the subset of genes with a 5'UTR and a Shine-Dalgarno-like signal predicted were classified as Shine-Dalgarno. Finally, the remaining genes where a Shine-Dalgarno-like signal was not detected were classified as UTR. The remaining genes for which a primary TSS was not detected but which were expressed at the whole transcriptome level were assigned to operons based on alignment and proximity to genes with a primary TSS. The maximum intergenic distance allowed for operon assignation was 500bp.

Genome-wide proteomics

Bacterial cell pellets were dissolved in lysis buffer containing 8 M Urea and 0.1% RapiGest (Waters) in 0.1 M ammonium bicarbonate buffer and were disrupted by applying two 40s cycles with FastPrep®-24 (MP Biomedicals). Protein concentration was determined using a BCA assay according to manufacturer's protocol (Thermo Fisher Scientific). Protein disulfide bonds were reduced by tris(2-carboxyethyl)phosphine (TCEP) and the resulting free cysteine residues were alkylated by iodoacetamide. Excessive iodoacteamide was captured by addition of N-acetyl cysteine. Extracted protein samples were diluted with ammonium bicarbonate buffer to reach a urea concentration of <2 M and then digested with sequencing-grade modified trypsin (Promega). To stop the tryptic digest and to precipitate RapiGest the pH was lowered to 2 using 50% trifluoro acetic acid (TFA). Water-immiscible degradation products of RapiGest were pelleted by centrifugation and the cleared peptide solution was desalted with C18 reversed-phase columns (Sep-Pak Vac C18, Waters), dried under vacuum, and re-solubilised to a final concentration of 1 mg/ml.

One µg of each peptide sample was analysed on a nano-LC system (Eksigent Technologies) connected to an LTQ Orbitrap XL mass spectrometer equipped with a nanoelectrospray ion source (Thermo Fisher Scientific). Peptides were separated on a fused silica microcapillary column (10 cm x 75 µm, New Objective) packed in-house with C18 resin (Magic C18 AQ 3 µm diameter, 200 Å pore size, Michrom BioResources) with a linear gradient from 95% solvent A (2% acetonitrile/0.1% formic acid) and 2% solvent B (98% acetonitrile/0.1% formic) to 35% solvent B over 90 min at a flow rate of

300 nl/min. The data acquisition mode was set to obtain one MS1 scan in the orbitrap at a resolution of 60,000 full width at half maximum followed by collision induced dissociation of the six most abundant precursor ions with a dynamic exclusion for 30 s. MS2 spectra were acquired in the linear ion trap.

Thermo raw files were converted into mzXML format using ProteoWizard. The acquired MS2 spectra were searched with OMSSA, XTandem, and MyriMatch against an Mtb H37Rv protein database (TubercuList v2.3, April 2011) additionally containing reversed sequences of all proteins in the database. Search parameters were as follows: semi-tryptic peptides (proteolytic cleavage after lysine and arginine unless followed by proline) and up to two missed cleavages were allowed, mass tolerance of the precursor ions was set to 20 ppm. Carbamidomethylation at cysteines was set as a fixed modification and oxidation at methionines as a variable modification. The output of the search engine was processed using PeptideProphet and iProphet. Only peptides at a false discovery rate of less than 1% were taken into consideration for further analysis. For MS1 based label-free quantification the openMS v1.8 framework was used and set up as described by (Weisser et al., 2013). Signals were normalised on peptide feature level such that the median signal in each sample is the same. Abundances of the three most intense peptides were averaged to get a protein abundance value. The same peptides were used for protein quantification across all samples and proteins with less than three peptides were included.

Statistical analysis

For functional enrichment analysis, GraphPad Prism v5.03c was used to compare the frequencies of different functional categories in respect to the H37Rv expressed transcriptome using two-tailed Chi-square tests. When multiple chi-square tests were performed, multiple testing correction was applied using the False Discovery Rate (FDR) method implemented in R. Non-parametric tests (Kruskal-Wallis or Mann-Whitney U tests) were used to evaluate differences among median levels of

expression. Protein quantification values were rescaled by dividing by 10^6. mRNA-protein correlations were determined using the Spearman rank coefficient.

Differential expression analyses

For whole transcriptome differential expression calling, genome coverage of reads mapping to genes, antisense and ncRNAs were used for statistical testing using DESeq (Anders and Huber, 2010), a method based on the negative binomial distribution and implemented in the R statistical environment. Differentially expressed genes were considered when fold changes between exponential growth and starvation were greater than or equal than 2-fold and the corresponding adjusted p-value was less than 0.01. For differential expression analysis of TSSs, the maximum number of reads mapped within a 50bp range from the TSS (peak height) were used for DESeq analysis.

Supplemental References

Kempsell, K.E., Ji, Y.E., Estrada, G., Colston, M.J., Cox, R.A. 1992. The nucleotide sequence of the promoter, 16S rRNA and spacer region of the ribosomal RNA operon of *Mycobacterium tuberculosis* and comparison with Mycobacterium leprae precursor rRNA. J. Gen. Microbiol. *138*, 1717-1727.

Noguchi, H., Taniguchi, T., Itoh, T., (2008). MetaGeneAnnotator: Detecting Species-Specific Patterns of Ribosomal Binding Site for Precise Gene Prediction in Anonymous Prokaryotic and Phage Genomes. DNA Res. *15*, 387-396.

Rice, P., Longden, I., Bleasby, A. (2000). EMBOSS: The European Molecular Biology Open Software Suite. Trends Genet. *16*, 276-277.
